# Prevalence of Substance Use in Northwest Syria: A Cross-Sectional Study

**DOI:** 10.3389/ijph.2026.1608611

**Published:** 2026-02-17

**Authors:** Isabel Draper, Dania Albaba, Heba Mesbah, Huda Taktak, Nidhal Saadoun, Mohammad Alhusein, Elharit Alissa, Anas Barbour, Zaher Sahloul, Nidal Moukaddam

**Affiliations:** 1 Psychiatry Department, Baylor College of Medicine, Houston, TX, United States; 2 MedGlobal, Rolling Meadows, IL, United States; 3 WHO, North West Syria Mental Health and Psychosocial Support MHSS0 Technical Working Group (TWG), Azzazz, Syria

**Keywords:** health services accessibility, internally displaced persons, syrian civil war, conflict region, substance use disorders

## Abstract

**Objectives:**

Syria has undergone significant socio-political turmoil since 2011 as internal conflict displaced portions of the population, destroyed infrastructure, and destabilized the economy. In the context of multilevel healthcare system disruptions, there have been increasing reports of substance use. This study seeks to evaluate patterns of substance use in Northwestern Syria.

**Methods:**

Trained staff administered questionnaires to community members in Azaz, Syria. Questions focused on the substance use prevalence, societal and public health impacts, and perspectives on treatment availability.

**Results:**

480 individuals were surveyed (80·88% male, 19·12 % female; 43·39% were 18–25 yrs, 11·5% reported personal substance use). H-booz (amphetamines), hashish (cannabis), and tramadol were the top 3 used. Displaced individuals exhibited twice the odds of substance use compared to non-displaced, with higher education and age demonstrating strong protective effects.

**Conclusions:**

The current sociopolitical and economic situation shaped patterns of substance use within Syria, with reported prevalence likely an underestimate given social desirability bias. Those commenting on their use met the criteria for substance use disorder. Results underscore the need for improved access to treatment options in the region.

## Introduction

From 2011 to 2024, the Syrian government and various factions fought. The conflict internally and externally displaced millions and injured or killed hundreds of thousands of civilians. Crucial infrastructure was destroyed, and remaining medical facilities struggle with a lack of staff, supplies, and funding [1]. The earthquakes in February 2023 further damaged the remaining infrastructure [2]. This destruction destabilized economic and social structures, limiting opportunities for engaging in traditional income-generating activities. This pattern is exemplified in Azaz, Syria, which is the geographical focus of this study. It is an area previously controlled by opposition forces with a population of 4·5 million; 2·9 million are internally displaced, and 3·7 million are food insecure [3]. Azaz’s population was 30,000 before the conflict, but an influx of internally displaced populations buoyed the number. At the time of the study, roughly 165,000 internally displaced people were estimated to be in Azaz, though these numbers may be outdated. In Syria, currency depreciation, high inflation, and commodity price increases have placed additional economic stress on a vulnerable population that may be forced to rely on alternative sources of income, including the production and/or sale of substances.

Substance use disorders (SUDs) include problematic use of substances like alcohol, nicotine, amphetamines, or other illicit materials. SUDs involve the use of these substances, which results in the impairment of an individual’s ability to function [4]. Development of tolerance and repeated failed attempts to quit constitute a cornerstone of the disorder. Medications, therapy, and neuromodulation can be used to treat SUDs. However, providing these treatments often necessitates the availability of specially trained healthcare workers and facilities. In countries with a history of significant conflict, internal displacement, and insufficient or destroyed medical infrastructure, there are complex risk factors and significant barriers to care affecting patterns of substance use. In Afghanistan, a nationally representative, cross-sectional survey found that the prevalence of any substance use was 5·03% with a sedative use prevalence of 6·71% [5]. Furthermore, individuals with previous experience of any traumatic event were more likely to use any substances compared to their peers. Those with depression, PTSD, and generalized anxiety disorder were also more likely to use any substance compared to their peers without psychiatric disorders. This association between a significant trauma burden and increased risk of substance use was similarly found in Lebanon, a country with the largest *per capita* number of refugees in the world, and its history of civil conflict and violence [6]. Furthermore, Palestinian refugees born in Lebanon had a higher lifetime rate of substance use than Palestinians and Syrians recently displaced from Syria [7]. The existing body of literature, while limited, suggests that refugees in countries in the Middle East with a history of conflict have an increased rate of substance use compared to non-displaced peers. This underscores potential associations between complex social factors, including trauma, conflict, displacement, and substance use.

Given the ongoing political and economic instability of Northwestern Syria, there has been a growing concern that substance use is rising. Seizures of amphetamines in the region reached a record high of 86 tons in 2021, double that of 2020 [8]. This suggests growing production and potential use of substances in the area. However, limited data is currently available on use patterns in Northwestern Syria. Previous studies sought to identify sources of drugs, the prevalence of use, demographics of users, reasons for the spread of drugs, and the impact of this spread on Syrians [9]. Previous efforts also sought to open and assess the need for an addiction treatment center in Azaz (opened with Physicians Across Continents (PAC) on 28 August 2022) and train Green Crescent Syria team members on mental healthcare and addiction. In the Afrin region, a study was conducted in collaboration with Turkish intelligence, the Activists’ Body, and National Army Factions to assess the prevalence of drugs and methods of trafficking between April 2021 and April 2022. A study in Damascus with Raba Al-Nahhas at the Syrian International Academy for Training and Development sought to analyze the impact of drug use and trafficking on homeless children. These previous studies could not fully elucidate substance use patterns due to methodology, scope, and structure issues. Developing an SUD unfolds as a complex interplay of personal, economic, and systemic socio-political factors, along with an individual’s social network and, in the aggregate, prevailing larger-scale conditions. This study seeks to elucidate patterns of substance use in Northwestern Syria and, subsequently, outline potential changes to increase care.

## Methods

Funding for this study was obtained through the World Health Organization (WHO), and MedGlobal, an international NGO, coordinated logistics. Institutional research approval was obtained from Baylor College of Medicine. This study employed a multi-pronged recruitment approach, utilizing questionnaires and focus group discussions to systematically gather insights from community members and service providers regarding 1- the prevalence of substance use and SUDs, 2-their societal and public health impacts, and 3- perspectives on treatment availability and obstacles. This paper summarizes the data from these questionnaires.

This study utilized the 2008 Field Guide and Rapid Assessment of Alcohol and Other Substance Use in Conflict-Affected and Displaced Populations used by the WHO [10] and United Nations High Commissioner for Refugees (UNHCR) [11]. The substance use thematic group in Northwest Syria worked on translating, developing, and adapting the survey forms from this guide to the Syrian context. While formal psychometric validation was not feasible due to field constraints, the adaptation process included expert review and pilot testing The questionnaire forms consisted of 92 questions covering demographics, socio-political issues, use patterns in the area, positive or negative perceptions regarding substance use, personal use, attempts to quit, the presence of IV drug use, the presence of HIV, and sexual relations due to substance use. All questionnaires were conducted in Arabic by trained personnel.

All subjects were recruited from Azaz, part of Aleppo’s governorate in Northwest Syria. MedGlobal organized the recruitment of participants in coordination with the PAC organization, the local health directorate, and other stakeholders.

A snowball sampling method was applied: Recruitment of community members was done by verbal invitations by NGO staff, followed by an email if indicated. We asked participants to reveal who else we may contact who might be willing to participate in the study.

Oral and written consent was obtained from all participants before enrollment in the research study. Names or identifying information were not collected. Questionnaires were administered in person by trained, Arabic-speaking staff. Before the study, staff received training in the appropriate protocol for administering the survey and recording participant responses. Staff administered the survey orally and recorded responses due to concerns for participant literacy differences affecting data collection. This paper summarizes the results of these surveys.

### Statistical Analysis

Statistical analysis was performed using STATA software (version 18.0; STATA Corp, College Station, Texas). The significance level for all statistical tests was set at α < 0.05. Continuous variables were summarized using means and standard deviations (SD) to describe the central tendency and variability for the descriptive statistics, including subject characteristics (such as the age of participants). Categorical variables were summarized using frequencies and percentages and analyzed using Pearson’s chi-square test.

We also performed multivariate logistic regression to examine predictors of substance use, including displacement status (non-displaced/displaced), education level, and age. Education was measured initially using multiple categories but was collapsed into three levels (low, medium, high) to improve model stability and interpretation. This categorization was based on theoretical relevance and sample size considerations, ensuring each group had sufficient observations for meaningful analysis.

$$\text{logit}\left(P\left(Y=1\right)\right)=\beta_{0}+\beta_{1}\text{Displaced}+\beta_{2}\text{Edu}\_\text{Medium}+\beta_{3}\text{Edu}\_\text{High}+\beta_{4}\text{Age}$$

The above model estimated adjusted odds ratios while controlling for all covariates simultaneously, with likelihood ratio tests and pseudo R^2^ values used to assess model fit. Predicted probabilities were calculated at the mean age to demonstrate how substance use risk varied across education levels and displacement status.

## Results

### Demographic Information

Four hundred eighty participants agreed to complete the questionnaire; 476 responses were included, and four were excluded due to missing or incomplete data. The sample size reflects a clear skew in male participants (80·88%) compared with female participants (Table 1). The average age of participants was 30.2 years, with an age range of 18–72 years. The majority of participants were under the age of 40 (83·2%). Approximately half of the respondents reported a high school diploma or lower level of education. Of those who agreed to disclose employment status, 42% were unemployed. Most participants refused to respond to questions about personal substance use history, with only 55 (11·45%) of participants endorsing personal use. However, the remaining 88·55% were willing to discuss details of substance use in the third person.

**TABLE 1:** Demographic information of participants (Azaz, Syria, 2023).

Category	Sub-category	Number (Percentage)
Gender	Total	476
	Male (n %)	385 (80·88%)
	Female (n %)	91 (19·12%)
Age	18–25	207 (43·49%)
	25–39	190 (39·92%)
	40–59	66 (13·87%)
	60–80	13 (2·73%)
Educational level	Elementary school	206 (43·28%)
	High school	55 (11·55%)
	College education	92 (19·33%)
	Advanced degree (graduate, MD, PhD)	112 (23·53%)
	None	11 (2·31%)
Occupation	Dependent/Family support	135 (28·36%)
	Unemployed	66 (13·66%)
	Agriculture/Farming	16 (3·36%)
	Construction/Building	23 (4·83%)
	Retail/Sales	68 (14·08%)
	Manufacturing/Industry	6 (1·26%)
	Freelance/Independent work	38 (7·98%)
	Business owners	26 (5·46%)
	Transportation/Driver	19 (3·99%)
	Education/Teacher	10 (2·10%)
	Student	1 (0·21%)
	Security/Military/Police	8 (1·68%)
	Healthcare/Medical	3 (0·63%)
	Technology/IT	9 (1·89%)
	Skilled trades/Craftsmen	26 (5·46%)
	Mechanic	6 (1·26%)
	Administrative	14 (2·94%)
	Cleaning/Sanitation	4 (0·84%)
Living situation	Alone	5 (1·04%)
	With family	422 (88·47%)
	With peers	47 (9·85%)
	At a hospital	3 (0·63%)

#### Descriptions of Personal Substance Use

The study explored the prevalence of substance use in two ways. The first is by reflecting community awareness - “Do you know somebody who uses drugs?” This was done to bypass stigma and attempt to reflect more accurately the prevalence of substance use. The second ascertains personal use. Discrepancies in answers highlight that many may have personal experiences but refused to divulge, as below:

Of the 54 (98·18%) who responded affirmatively to whether they had used any substance or alcohol, 11 (20·37%) reported that life stressors, poverty, trying new things, having fun, pain control after a war injury, and bad company may be contributing factors to their substance use. Individuals who denied substance use stated mainly that they did not know the reason for use (418, 99·29%). Of those who reported never using a substance, 3 (0·76%) cited reasons for use: used to have fun (2, 0.48%) or used because of life stressors (1, 0·24%). One participant who had previously denied using substances responded “yes” when asked about intravenous substance use. These discrepancies are noted here to provide further context to community substance use, note the complexity of the topic being discussed, and explain the rationale for including these contradicting responses.

### Specific Substance Use

Participants were asked about the use of specific substances in their community and, if applicable, their personal use.

Substances were classified by and ranked within categories based on frequency. The total number of participants responding to this question was 436 out of the 480 who agreed to complete the questionnaire (Table 2). Percentages for use frequency were calculated based on the number of mentions of each substance being used in the community from these 436 respondents. Tramadol (20·04%), H Booz (24·52%), and Hashish (Cannabis) (22·79%) were the three substances with the highest rate of use.

**TABLE 2:** Participants were asked, “What substances are being used in the community?” (Azaz, Syria, 2023).

Substance		N (%)
Cannabis	Hashish	224 (22·79%)
Opioids	Tramadol	197 (20·04%)
	Morphine	15 (1·53%)
	Opium	8 (0·81%)
	Heroin	5 (0·51%)
Sedative- hypnotic	Benzodiazepines	37 (3·76%)
	Biogabalin (pregabalin)	28 (2·85%)
	Diprivian	5 (0·51%)
Inhalants	Gasoline	4 (0·41%)
Stimulant	H booz (crystal meth)	241 (24.52%)
	Captagon	132 (13.43%)
	Cocaine	10 (1.02%)
	Amphetamines	9 (0.92%)
	Hallucinogens	2 (0.2%)
Other	Alcohol	16 (1.63%)
	Tobacco	20 (2.03%)
	Bird feed (qumbuz)	10 (1.02%)

Given much discussion regarding poverty’s impact on Northwest Syria, identifying the price ranges of the substances used was of special interest. Users largely reported that there was price variation. Notably, the three most self-reported used substances in this region were Hashish, H Booz, and Tramadol at 21·7%, 21·6%, and 18·8%, respectively. Cannabis was reported to be priced at about $30–60 per 400 g, and H. Booz at $20–30 per gram. Tramadol was reported to be $4-6 per pill pack (Table 3). The most expensive substance of use was cocaine. It is important to note that exact prices may have changed due to fluctuations in the market over time. Variations in prices were largely unknown to non-users (99·53%). This information, albeit from a small group of users, is valuable for demonstrating the relative pricing of substances.

**TABLE 3:** “How much is each substance?” (Azaz, Syria, 2023).

Substance		Price range (in USD)	
Cannabis/Hashish		$30–60	per 400 g
Opioids	Tramadol	$4–6	per pillpackbox
	Opium (afyum)	$0·04	per ampule
	Heroin	$40–100	per gram
	Morphine	$4–7	per ampule
Sedative- hypnotic	Diazepam	$2–10	per ampule
	Lorazepam (zolam)	$7–10	per ampule
	Pregabalin	$2–5	per pillpackbox
	Gabapentin	Unspecified	​
Inhalants	Gasoline	Unspecified	​
Stimulants	Captagon	$0·20–1	per pill
	H-booz	$20–30	per gram
	Cocaine	$20–100	per gram
	Cocaine “stone”	$2000	per gram
	Cocaine powder	$3000	per gram
	Ecstasy	$1–3	per pill
Alcohol		$5–10	per drink
Tobacco		Unspecified	​
Others	SSRIs (sertraline, paroxetine)	$2–5	per pillpackbox
	Bird feed قمبز	$10	per kilogram
	Propofol (diprivan)	$8–10	per ampule
	“Relief” (diclofenac, paracetamol, cetirizine)	$1.50	per pillpackbox
	Codeine	$2	per pillpackbox

#### Intravenous Drug Use

Among the fifty-four participants who reported personal substance use overall, the majority, fifty (98·04%), reported intravenous drug use.

#### Substance-Related Problems

80% reported facing issues due to their substance use. Six participants (11·76%) who had responded earlier that they were not using later reported facing problems because of use.

Similar discrepancies were noted when queried about attempts to quit substance use: Of those who had previously denied substance use, 13 answered yes. When asked about previous attempts to leave, 305 non-users responded “no” and 32 (59·26%) users responded “yes,” 21 (38·88%) users responded “no,” and one user (1·8%) provided no answer.

#### Factors Involved in Quitting

Most did not respond, and those who did gave vague answers such as “many times.” Obstacles to relapse were described similarly amongst users and non-users: depression or life stressors, absence of specialized centers to help, peer pressure, and husbands forcing wives to use. Users additionally mentioned withdrawal symptoms. Regarding help from a qualified person or place, 34% of users reported having used medical/specialized help to detox/quit, compared to 3% of non-users (Table 4). Of note, seven respondents reported receiving help from rehabilitation centers but had previously denied use.

**TABLE 4:** “Where do you receive help from if you want to quit?” (Azaz, Syria, 2023).

Help source	Family members	Specialized centers for addiction	Medical personnel	Friends	Community leaders	I do not get help as I am not using	No answers	I do not know
Non-user	0	7 (36·84%)	0	4 (80%)	1 (100%)	334 (97·38%)	46 (70·77%)	29 (87·88%)
User	4 (100%)	12 (63·16%)	4 (100%)	1 (20%)	0	9 (2·62%)	19 (29·23%)	4 (12·12%)

#### Quality of Available Rehabilitation Centers

41 participants responded that the services provided were good. Ten believed that they promoted awareness in the community, only two received agonist therapy, and one reported clean needle provision. Healthy food and privacy/confidentiality were described as positives. A minority of respondents questioned staff qualifications.

#### Testing for HIV

Less than 1% of non-users reported HIV testing. 5·5% of users reported being tested for HIV. Seven participants (1·4%) reported receiving counseling after obtaining HIV results.

#### Suggestions for Preventing Substance Use

Respondents emphasized the following as potentially helpful-increasing awareness (23·43%), avoiding being around users (20·08%), being away from environments encouraging use (20·08%), increasing resources for treatment (8·79%), improving law enforcement, controlling borders, and arresting drug dealers (8·58%), increasing family support and religious commitments (7·95%), financial support and employment opportunities (7·74%) (Table A1).

#### Predictors of Use

Within this sample, displaced individuals had twice the odds of substance use compared to non-displaced individuals (aOR = 2.01, 95% CI: 1.04–3.89). Higher education was strongly protective, associated with 80% lower odds of substance use versus low education (aOR = 0.20, 95% CI: 0.10–0.43). Each additional year of age reduced the odds by 8% (aOR = 0.92, 95% CI: 0.88–0.96). Predicted probabilities showed displaced individuals with low education had the highest risk (26.4%), while non-displaced individuals with high education had the lowest (3.5%). These findings suggest that displacement increases substance use risk, but higher education may mitigate this effect, particularly among younger populations.

## Discussion

This study provides comprehensive results about the demographic patterns of substance use within Azaz, an area whose composition is representative of Northwest Syria, where a confluence of political, environmental, and social disruptions has contributed to a rise in substance use. These results demonstrate a high prevalence of substance use, with the most common substances used being H-booz (methamphetamines), Captagon, Hashish, and Tramadol. Findings suggest that users predominantly use alone and are the only users in their household. While a small percentage of the sample acknowledged personal substance use, a larger percentage of the sample responded in detail about community use in such a way that suggests a significant level of awareness and, potentially, unreported personal use. This discrepancy demonstrates the impact of non-response and social desirability bias on this data set.

Additionally, this discrepancy further reinforces the social stigma surrounding substance use and potential barriers to accessing treatment for SUDs. Further surveys should explore this finding as a possible cause of a discrepancy noted in our results: non-users detailed reporting information about substance use. The types of substances used do not align fully with news/media reports of captagon use as the primary substance, but rather highlight more prevalent methamphetamine (hbooz) and hashish use. There are two ways to view our results, and the authors presume stigma surrounding substance use may be involved: looking strictly at those who reported personal use, rates are congruent with those in other countries. However, when information about substance patterns is compiled regardless of the user/non-user dichotomy, the picture obtained is that of a population with a high SUD burden.

Furthermore, analysis of the predictors of use within this recruited sample revealed patterns of vulnerabilities consistent with trends observed globally, particularly in countries like Afghanistan and Lebanon [5, 7]. Forced migration consistently emerges as a risk factor for substance use [12, 13]. Similarly, the strong protective role of education reinforces cross-cultural evidence that higher educational attainment serves as a buffer against SUDs across various populations [14, 15]. The pronounced risk gradient between displaced individuals with limited education and their higher-educated, non-displaced counterparts reflects the intersectional vulnerabilities documented internationally and in this sample, suggesting that the displacement amplifies existing disparities in substance use patterns across diverse sociopolitical contexts.

The vast majority (98.04%) of those acknowledging substance use reported intravenous drug use, potentially highlighting that milder levels of SUDs are less reported. However, a limited proportion had been tested for bloodborne pathogens like HIV; there is a noted discrepancy between reported counseling and testing for HIV. In light of how many users in our sample reported IV drug use, the lack of counseling and testing is alarming. This information stresses the importance of public health interventions targeted at intravenous drug users, including increased information about the potential for blood-borne diseases like HIV and safe needle use.

The lack of accessibility to treatment for substance use is highlighted by several findings of our questionnaire, including the lack of specialized centers for SUD treatment or medical doctors and the reliance on community or religious leaders who may not have specialized training for SUD treatment and prevention. As mentioned above, the lack of diagnostic options and care for co-occurring disorders and exposure to previous traumatic experiences is highlighted by questionnaire results, as users and non-users report that unresolved issues/trauma are antecedents to SUD. Even without available treatment, those who are using are reflecting on reasons for use, which indicates the severity of their use, as this represents an aspect of the pre-contemplative mindset for stopping or changing substance use patterns. Additionally, given the high rate of use of tramadol in the region, treatment options available should ideally include medication-assisted treatment (MAT). MAT is more efficacious than therapy alone [16]. However, MAT requires a functional medical infrastructure for initiating medication and maintaining long-term monitoring. In Northwestern Syria, there is currently limited or no access to MAT for substance abuse disorders.

Reported suggestions for improvement include expanding on themes found in this study and repeating this study, given recent changes in the sociopolitical situation in Syria. The end of the civil war represents an opportunity to repeat this survey to generalize better use patterns nationwide. Questions concerning vulnerable populations like women and children should be added to ascertain connections between substance use, conflict, and human trafficking. Due to the high reported rate of intravenous drug use, future studies may investigate how users acquire their needles and if any measures are taken to ensure that these supplies are safe. For this type of study, bilingual fluency is required. Therefore, for the reproduction and expansion of these findings, both linguistic and cultural fluency are a requirement.

There is a general lack of awareness of systemic factors' influence on SUD in the questionnaire. A plethora of published reports describe associations of substance trafficking with war and human trafficking, but our questionnaire found mostly awareness of financial issues and “bad company”/interpersonal influences. Prevention and treatment programs should carefully consider the balance of personal versus systemic interventions and the limitations of person-focused treatments in areas with such high socio-political conflict.

Limitations to this study include the nature of the data collected, the difficulty in obtaining information about individuals' substance use, social desirability bias, and significant barriers to recruiting participants. The data collected was primarily descriptive, including the characteristics of those responding to the questionnaire and their shared observations and experiences with substance use in the community.

The majority of individuals refused to answer questions regarding personal substance use history or whether they knew others who used substances. This may have been due to social desirability bias- SUDs remain difficult to discuss or acknowledge, and this attitude was evident beyond the individual level. Due to stigma, universities did not allow students to be recruited or surveyed on their campuses, which altered initial recruitment plans. Questionnaires had to be held in other settings with the assistance of the local health government. This suggests that there was a significant element of stigma that may have prevented participation in the study and deterred participants from being forthcoming about the true nature of substance use in the region. There was also a significant skew in the number of individuals who could be recruited and consented to participate in this study: the majority were male. As 480 participants were recruited, this is the most extensive study investigating patterns of substance use in Northwestern Syria [17].

## Appendix

**TABLE A1:** “What could help abstain from using substances?”. (Azaz, Syria, 2023).

What could help?	Non-user	User
Raise awareness about the dangers of substance use	104 (92.86%)	8 (7.14%)
Financial support (employment to provide for the family)	21 (56.76%)	16 (43.24%)
Avoid negative influences and individuals who use substances	78 (82.11%)	7 (17.89%)
Increase the number of rehabilitation centers and qualified personnel	37 (88.10%	5 (11.90%)
Strengthen family support and enhance religious commitment	38 (100%)	0
Enhance law enforcement efforts	36 (87.80%)	5 (12.20%)
Prevent displacement	0	1
I do not know	94 (97.92%)	2 (2.08%)
No answer	13 (100%)	0
Total	422	54
